# Clinical Profile, Prognostic Factors, and Outcome Prediction in Hospitalized Patients With Bloodstream Infection: Results From a 10-Year Prospective Multicenter Study

**DOI:** 10.3389/fmed.2021.629671

**Published:** 2021-05-20

**Authors:** Longyang Jin, Chunjiang Zhao, Henan Li, Ruobing Wang, Qi Wang, Hui Wang

**Affiliations:** Department of Clinical Laboratory, Peking University People's Hospital, Beijing, China

**Keywords:** bacterial bloodstream infection, mortality, pathogenic spectrum, prediction model, prognostic factors

## Abstract

**Background:** Bloodstream infection (BSI) is one of the most common serious bacterial infections worldwide and also a major contributor to in-hospital mortality. Determining the predictors of mortality is crucial for prevention and improving clinical prognosis in patients with nosocomial BSI.

**Methods:** A nationwide prospective cohort study was conducted from 2007 until 2016 in 16 teaching hospitals across China. Microbiological results, clinical information, and patient outcomes were collected to investigate the pathogenic spectrum and mortality rate in patients with BSI and identify outcome predictors using multivariate regression, prediction model, and Kaplan–Meier analysis.

**Results:** No significant change was observed in the causative pathogen distribution during the 10-year period and the overall in-hospital mortality was 12.83% (480/3,741). An increased trend was found in the mortality of patients infected with *Pseudomonas aeruginosa* or *Acinetobacter baumannii*, while a decreased mortality rate was noted in *Staphylococcus aureus*-related BSI. In multivariable-adjusted models, higher mortality rate was significantly associated with older age, cancer, sepsis diagnosis, ICU admission, and prolonged hospital stay prior to BSI onset, which were also determined using machine learning-based predictive model achieved by random forest algorithm with a satisfactory performance in outcome prediction.

**Conclusions:** Our study described the clinical and microbiological characteristics and mortality predictive factors in patients with BSI. These informative predictors would inform clinical practice to adopt effective therapeutic strategies to improve patient outcomes.

## Introduction

Despite the great advances in medical diagnosis and therapy over the past decades, bloodstream infection (BSI) remains a major cause of infectious disease morbidity and mortality in both low- and middle- or high-income countries (1, 2). Several studies have reported that BSI was the seventh most common cause of death and the leading cause of death caused by infections (1, 2). It is estimated that at least 23 per 100,000 people die each year shortly following an episode of BSI (2). Immunocompromised, chemotherapies, intravascular catheters, and high consumption of antibiotics rendered hospitalized patients highly vulnerable to bacterial colonization, local infection, and even systemic infection (3). A previous study showed that different bacterial species had a significant impact on the prognosis of bacteremia, but the pathogenic spectrum responsible for BSI varied substantially over time and by region (4). Moreover, bloodstream infection can lead to sepsis, an extreme systemic response to infection, which is associated with increased mortality and length of hospital stay and additional medical costs (5). Previous efforts have demonstrated that rapid assessment and intervention is crucial for the prognosis of BSI patients, especially in the emergency department and ICU, because implementing timely and effective infection treatment can significantly reduce the incidence of BSI-associated deaths (6, 7).

Accurate identification of predictors associated with mortality in patients with BSI is critical to informing clinical interventions and improving clinical outcomes. Although some prognostic factors have been identified as potential predictors for BSI mortality, most previous reports particularly focused on a single group of people, such as children or the aged, or with specific clinical conditions including cancer and trauma as well as causative organisms equipped with multidrug resistance (8–11). In addition, multiple machine learning approaches have been developed and increasingly used in predicting unfavorable outcomes and in identifying predictors of mortality for different types of disease, with better performance than the classical multivariate regression analysis method (12). Previous studies have explored the use of the random forest model, one of the machine learning approaches, in the prediction of multidrug-resistant bacterial infection and mortality due to sepsis in the emergency department and in providing significant clinical outcome predictors based on permutation importance of different variables (13, 14). Until now, however, very few studies have characterized the feasibility of machine learning technology for the purpose of predicting all-cause mortality of hospitalized patients with BSI.

In the present study, we sought to describe the trends of the incidence of key bloodstream pathogens and BSI-associated mortality over time for the period 2007–2016, which were collected by a national prospective surveillance program. Independent factors for all-cause mortality in hospitalized patients with BSI were also assessed. These results might facilitate a physician's decision-making process concerning rational treatment for high-risk individuals with bacteremia and optimize clinical resources.

## Materials and Methods

### Study Design

This study was an investigative and predictive analysis based on BSI patients' clinical data from the CARES study (Chinese Antimicrobial Resistance Surveillance of Nosocomial Infections), which is a nationwide, longitudinal, prospective study encompassing 16 tertiary-care teaching hospitals in 10 provinces in China between 2007 and 2016 (15–17). Each hospital has at least 1,200 beds and one infectious disease department and infection control committee with specialist doctors, nurses, and microbiological laboratory personnel. Clinicians can be informed immediately by telephone with the positive blood culture as a critical value. Our aim in this study was to analyze the pathogenic spectrum of bacteremia and further elucidate the independent predictors for all-cause mortality at 28 days among hospitalized patients with BSI. Cases were eligible for this study if they had a positive blood culture for gram-negative or gram-positive bacteria and sufficient documentation in the electronic health records to assess therapy and outcomes within 28 days of the positive blood culture. This study was approved by the Research Ethics Board at Peking University People's Hospital, which waived the need for informed consent, because of the observational nature of the study.

### Clinical Data Collection

All cases considered in this study were hospitalized patients aged ≥18 years and had at least one documented isolation from positive blood culture during their hospitalization. In order to identify the clinical predictors of BSI mortality, each patient was included only once at the time of the first bacterial isolation from blood culture during the study period. All elements in demographic data, antibiotic administration records, laboratory and microbiological results, and clinical information, including potential predictors for mortality, were extracted from electronic health records (EHRs) by trained reviewers. The primary outcome variable was in-hospital mortality within the first 28 days after drawing the positive blood cultures. Additionally, patients with missing observations or treated on an outpatient basis were excluded retrospectively. All bloodstream isolates were transferred to a reference laboratory (Peking University People's Hospital) and identified by matrix-assisted laser desorption/ionization time-of-flight mass spectrometry. Cases with coagulase-negative *Staphylococcus* isolated from a single blood culture without any clinical evidence of infection were also excluded.

### Prognostic Factor Analysis

Cox multivariable regression analysis was performed to identify independent predictors for BSI 28-day mortality. We conducted univariate logistic regression analysis for each candidate variable using Pearson's chi-square test or Fisher's exact test, with a *P* < 0.10 being the criterion for further analysis in the backward, conditional stepwise multivariable regression model. The Hosmer–Lemeshow goodness-of-fit test was used to assess model fit. Hazard ratios, 95% confidence intervals, and associated *P*-values were also reported.

### Statistical Analysis

For univariate analysis, normally distributed continuous variables were expressed as means ± standard deviations (SD) and compared using *t*-test or Mann–Whitney *U* test. Categorical variables and their relative frequencies were expressed as absolute numbers and compared using Pearson's chi-square test or Fisher's exact test. The multivariate regression analyses were performed to identify independent predictors using IBM SPSS software (version 24.0) for Windows. The chi-square test for trend in proportions was performed to determine significant variations in etiology and mortality during the study period. All reported *P*-values are two-sided and statistical significance was set as *P* < 0.05. In addition, all cases and potential factors were used to develop the random forest model and export strong predictors for BSI-related mortality using the R package randomForest. We also quantified the discriminative performance using the area under the ROC curves (AUC), sensitivity, specificity, positive predictive value, and negative predictive value. For variables significantly associated with mortality in both multivariate analysis and random forest model, a Kaplan–Meier curve was plotted to show the survival probabilities at 28 days.

## Results

### Distribution and Incidence of Bacteremia Isolates

From 2007 to 2016, 4,708 patients with positive blood culture were documented in 16 tertiary-care teaching hospitals, and each first bacterial isolate was enrolled in microbiology analysis. Among these isolates, the proportion of gram-negative isolates was higher than that of gram-positive ones (70.33 vs. 29.67%). Overall, *Escherichia coli* (29.21%, 1,375/4,708) and *Klebsiella pneumoniae* (12.70%, 598/4,708) were the most common BSI-causing pathogens followed by *Staphylococcus aureus* (9.79%, 461/4,708), *Acinetobacter baumannii* (7.03%, 331/4,708), and *Pseudomonas aeruginosa* (6.33%, 298/4,708). Notably, *E. coli, K. pneumoniae, S. aureus*, and *A. baumannii* remained predominant as the top 4 pathogens responsible for BSI during the study period except *P. aeruginosa* that substituted *A. baumannii* in 2009–2010. The 10 most common pathogens are listed in Table 1 by a 2-year period.

**Table 1:** Distribution and incidence of bloodstream bacterial isolates during the study period.

**Rank**	**Isolates (%) during**					
	2007–2008 (1,080^*^)	2009–2010 (1,061^*^)	2011–2012 (808^*^)	2013–2014 (866^*^)	2015–2016 (893^*^)	Ten years (4,708^*^)
1	*E. coli* (29.07)	*E. coli* (26.58)	*E. coli* (30.07)	*E. coli* (30.72)	*E. coli* (30.24)	*E. coli* (29.21)
2	*K. pneumoniae* (11.57)	*K. pneumoniae* (10.65)	*K. pneumoniae* (13.99)	*K. pneumoniae* (15.13)	*K. pneumoniae* (12.99)	*K. pneumoniae* (12.70)
3	*S. aureus* (10.83)	*S. aureus* (10.08)	*S. aureus* (10.02)	*S. aureus* (8.55)	*S. aureus* (9.18)	*S. aureus* (9.79)
4	*P. aeruginosa* (6.02)	*P. aeruginosa* (7.45)	*A. baumannii* (8.91)	*A. baumannii* (7.27)	*A. baumannii* (8.06)	*A. baumannii* (7.03)
5	*E. faecium* (5.46)	*A. baumannii* (6.41)	*P. aeruginosa* (6.31)	*S. epidermidis* (5.54)	*P. aeruginosa* (6.16)	*P. aeruginosa* (6.33)
6	*S. epidermidis* (5.37)	*S. hominis* (5.37)	*S. epidermidis* (6.06)	*P. aeruginosa* (5.54)	*E. faecium* (4.82)	*S. epidermidis* (4.91)
7	*A. baumannii* (5.19)	*E. faecium* (4.81)	*E. faecium* (4.58)	*E. faecium* (4.27)	*S. hominis* (4.37)	*E. faecium* (4.82)
8	*S. hominis* (3.98)	*S. epidermidis* (4.52)	*E. cloacae* (4.08)	*E. cloacae* (3.35)	*E. cloacae* (3.92)	*S. hominis* (3.99)
9	*E. cloacae* (2.59)	*E. cloacae* (4.15)	*S. hominis* (3.34)	*E. faecalis* (2.54)	*S. epidermidis* (3.14)	*E. cloacae* (3.59)
10	*E. faecalis* (2.59)	*E. faecalis* (2.83)	*E. faecalis* (2.85)	*S. hominis* (2.54)	*E. faecalis* (2.69)	*E. faecalis* (2.70)

* *Number of isolates*.

### Demographic and Clinical Characteristics

**Figure 1:**
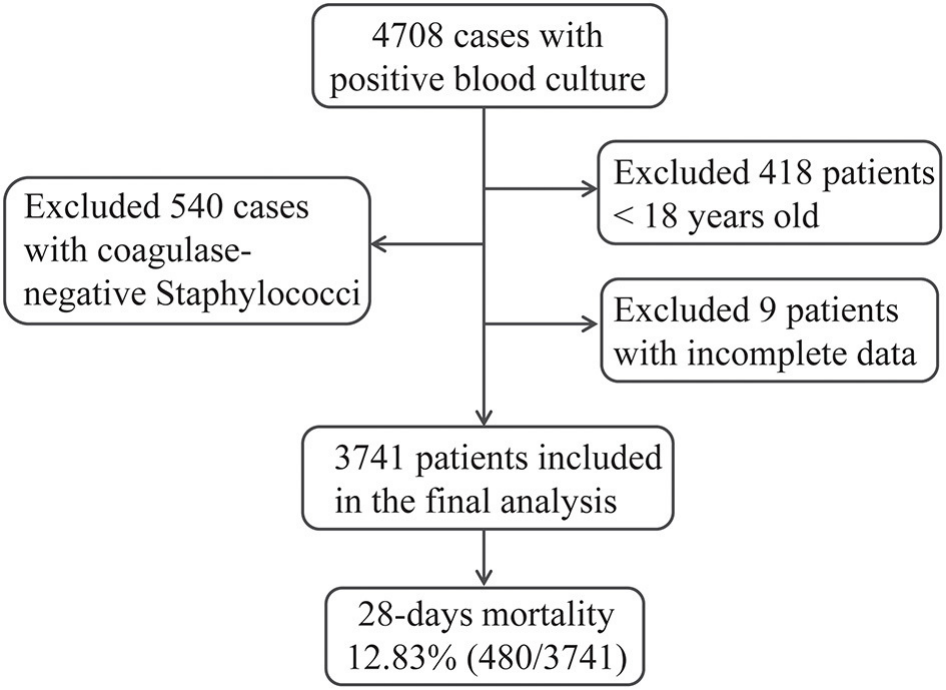
Flowchart for the inclusion of patients eligible to this study.

**Figure 2:**
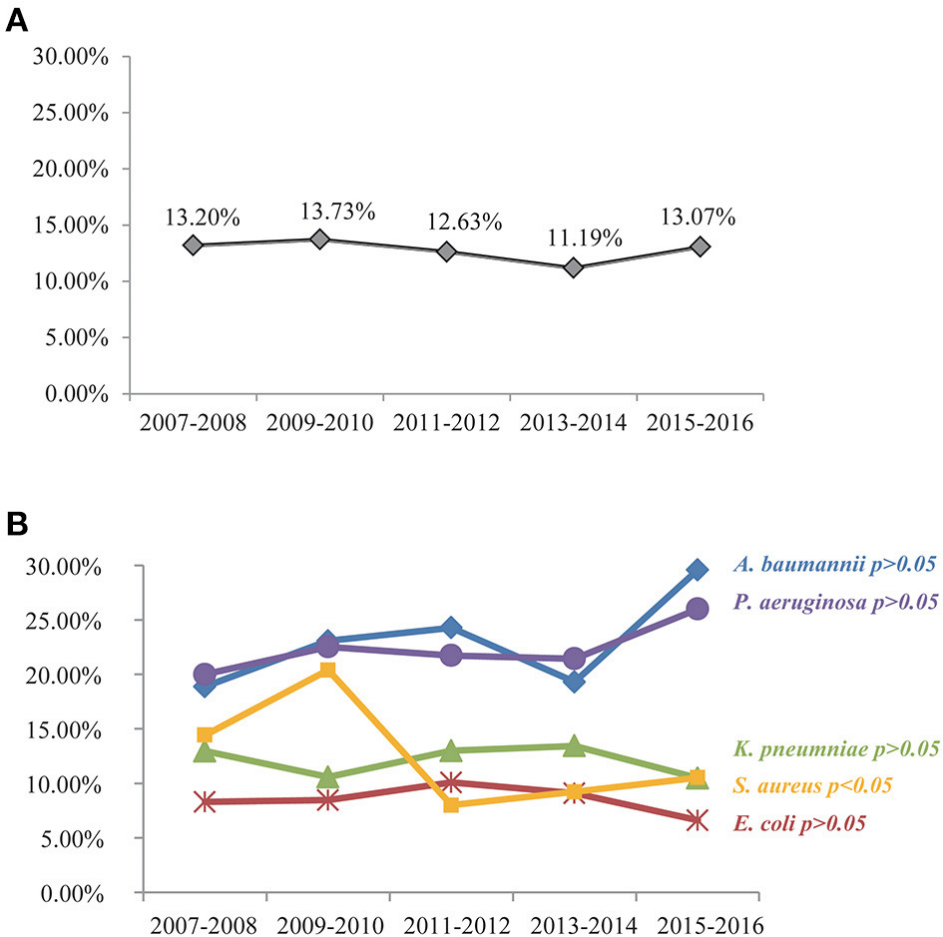
All-cause mortality among patients with nosocomial BSI by a 2-year period **(A)** and 10-year trend in mortality related to different causative bacteria **(B)**. *S. aureus*-related mortality rate has dropped significantly during the 10-year period (*P* < 0.05). Upward trends in *A. baumannii*- and *P. aeruginosa*-related mortalities were noted but with no statistical significance as assessed by the chi-square test (*P* > 0.05). *E. coli*- and *K. pneumoniae*-associated mortality remained stable and relatively lower (*P* > 0.05).

For the purpose of identifying predictors of BSI-associated mortality in the present study, a total of 3,741 hospitalized patients fulfilled our inclusion criteria and were included in the final analysis (Figure 1). The overall 28-day mortality rate was 12.83% (480/3,741) during the 10-year study period and did not vary significantly among different years (Figure 2A). However, the BSI-associated mortality varied somewhat over time by different causative organisms. Mortality due to *S. aureus*-related BSI declined from 20.39% in 2009–2010 to < 10% in 2015–2016, while that associated with *A. baumannii* and *P. aeruginos*a increased between the years 2007–2008 and the years 2015–2016. *E. coli*- and *K. pneumoniae*-associated mortality remained stable and relatively lower than that of other bloodstream isolates (Figure 2B).

Demographic and clinical characteristics of bacteremia patients as well as the results of univariate analysis of the comparison between survived and died groups are shown in Table 2. The mean age of all BSI cases was 56 years (SD = 17.33, range = 18–99) and patients were predominantly male (60.09%). The most common underlying condition was malignancy (28.82%) and the source of the BSI was primary (unknown origin) in 57.04% of the cases. Mortality varied according to comorbidities, type of catheter, and clinical therapy. The highest mortality was accompanied with sepsis symptoms (25.74%) and ICU admission (24.25%). Multiple statistically significant predictors (*P* < 0.05) were identified in the univariate analysis. Compared with survived patients, dead patients with BSI were more likely to be >65 years of age, their length of hospital stay prior to BSI was >14 days, sepsis was present, intermittent temperature was <35 or >40°C, they were admitted to the ICU, and inappropriate empirical treatment was provided.

**Table 2:** Demographics, comorbidities, and clinical treatments of patients with BSI.

**Variables**	**Total, *N* = 3,741 (100%)**	**Survived, *N* = 3,261 (87.17%)**	**Died, *N* = 480 (12.83%)**	***P*-value**
**Demographics**				
Age >65 years	1,188 (31.76%)	948 (79.80%)	240 (20.20%)	<0.001
Male	2,248 (60.09%)	1,943 (86.43%)	305 (13.57%)	0.098
**Comorbidity**				
Malignancy	1,078 (28.82%)	922 (85.53%)	156 (14.47%)	0.056
Diabetes mellitus	601 (16.06%)	513 (85.36%)	88 (14.64%)	0.147
Hypertension	522 (13.95%)	461 (88.31%)	61 (11.69%)	0.399
Cardiovascular disease	607 (16.23%)	515 (84.84%)	92 (15.16%)	0.061
Cerebrovascular disease	359 (9.60%)	304 (84.68%)	55 (15.32%)	0.138
Liver disease	521 (13.93%)	445 (85.41%)	76 (14.59%)	0.196
Renal disease	422 (11.28%)	365 (86.49%)	57 (13.51%)	0.659
Respiratory disease	452 (12.08%)	383 (84.73%)	69 (15.27%)	0.099
Immunocompromised	34 (0.91%)	27 (79.41%)	7 (20.59%)	0.174
**Invasive procedures**				
Central venous catheter	591 (15.08%)	503 (85.11%)	88 (14.89%)	0.103
Peripheral intravenous catheter	2,978 (79.60%)	2,608 (87.58%)	370 (12.42%)	0.142
Urinary catheter	1,114 (29.78%)	955 (85.73%)	159 (14.27%)	0.086
Invasive mechanical ventilation	204 (5.45%)	179 (87.75%)	25 (12.25%)	0.800
Surgery within the past 14 days	975 (26.06%)	833 (85.44%)	142 (14.56%)	0.060
**Condition prior to infection onset**				
Preinfection length of stay >14 days	1,139 (30.45%)	933 (81.91%)	206 (18.09%)	<0.001
Prior hospitalization within 90 days	1,245 (33.28%)	1,069 (85.86%)	176 (14.14%)	0.092
Antibiotic exposure within 2 months	1,685 (45.04%)	1,485 (88.13%)	200 (11.87%)	0.111
**Primary site of infection**				
Urinary tract	305 (8.15%)	276 (90.49%)	29 (9.51%)	0.070
Respiratory system	351 (9.38%)	297 (84.62%)	54 (15.38%)	0.133
Central line-associated	224 (5.99%)	187 (83.48%)	37 (16.52%)	0.089
Intra-abdominal	587 (15.69%)	510 (86.88%)	77 (13.12%)	0.821
Skin and soft tissue	140 (3.74%)	125 (89.29%)	15 (10.71%)	0.445
Unknown	2,134 (57.04%)	1,866 (87.44%)	268 (12.56%)	0.566
**Clinical symptoms**				
Presentation with sepsis	773 (20.66%)	574 (74.26%)	199 (25.74%)	<0.001
Temperature <35 or >40°C	407 (10.88%)	336 (82.56%)	71 (17.44%)	0.003
**Treatments**
ICU admission	862 (23.04%)	653 (75.75%)	209 (24.25%)	<0.001
Immunosuppressive therapy	394 (10.53%)	337 (85.53%)	57 (14.47%)	0.305
Inappropriate empirical treatment	760 (20.32%)	640 (84.21%)	120 (15.79%)	0.006

### Independent Mortality Predictors

All variables that were statistically significant (*P* < 0.10) in the univariate analysis were included in further Cox regression analysis. In the multivariate analysis, factors independently associated with higher mortality in patients with BSI included age >65 years (HR, 2.219; 95% CI, 1.745–2.822; *P* < 0.001), malignancy (HR, 1.301; 95% CI, 1.005–1.683; *P* = 0.046), pre-infection length of stay >14 days (HR, 1.615; 95% CI, 1.271–2.052; *P* < 0.001), ICU admission (HR, 6.261; 95% CI, 1.859–21.079; *P* < 0.001), and presentation with sepsis (HR, 3.973; 95% CI, 3.202–4.929; *P* < 0.001).

Besides, we also identified potential predictors with the highest coefficients based on permutation importance using the random forest algorithm. It is found that ICU admission [variable importance (VI), 53.89], presentation with sepsis (VI, 21.66), age >65 years (VI, 11.58), inappropriate empirical treatment (VI, 9.29), temperature < 35 or >40°C (VI, 8.92), pre-infection length of stay >14 days (VI, 8.32), malignancy (VI, 5.62), cardiovascular disease (VI, 4.42), surgery within the past 14 days (VI, 4.28), and central line-associated (VI, 3.31) were the top 10 important predictors in the random forest model. These results were consistent with what is found in the multivariate analysis.

**Figure 3:**
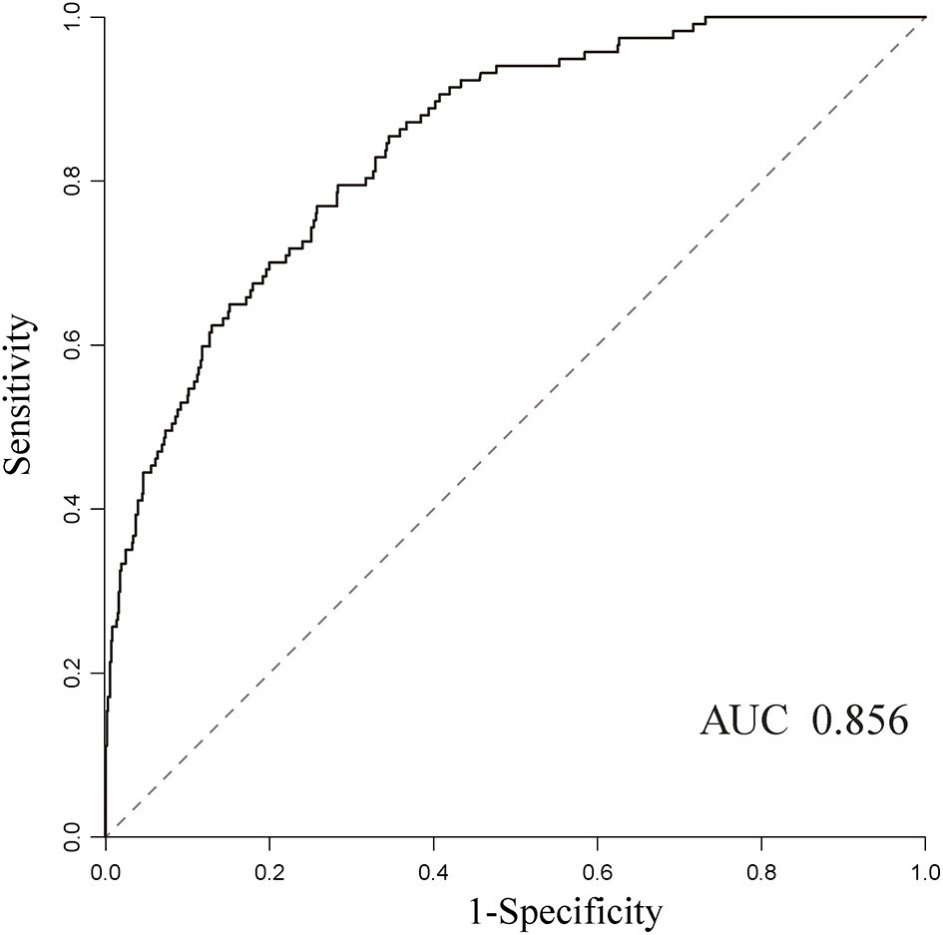
The receiver operating characteristic curves for predicting mortality using the random forest algorithm and the AUC was 0.856.

In addition, we also evaluated the model's ability to discriminate outcome based on all clinical data collected. The sensitivity (0.81), specificity (0.74), negative predictive value (0.95), and AUC (0.856) showed high to moderate predictive performance, while the positive predictive value is only 0.32 (Figure 3).

### Survival Curve Analysis

**Figure 4:**
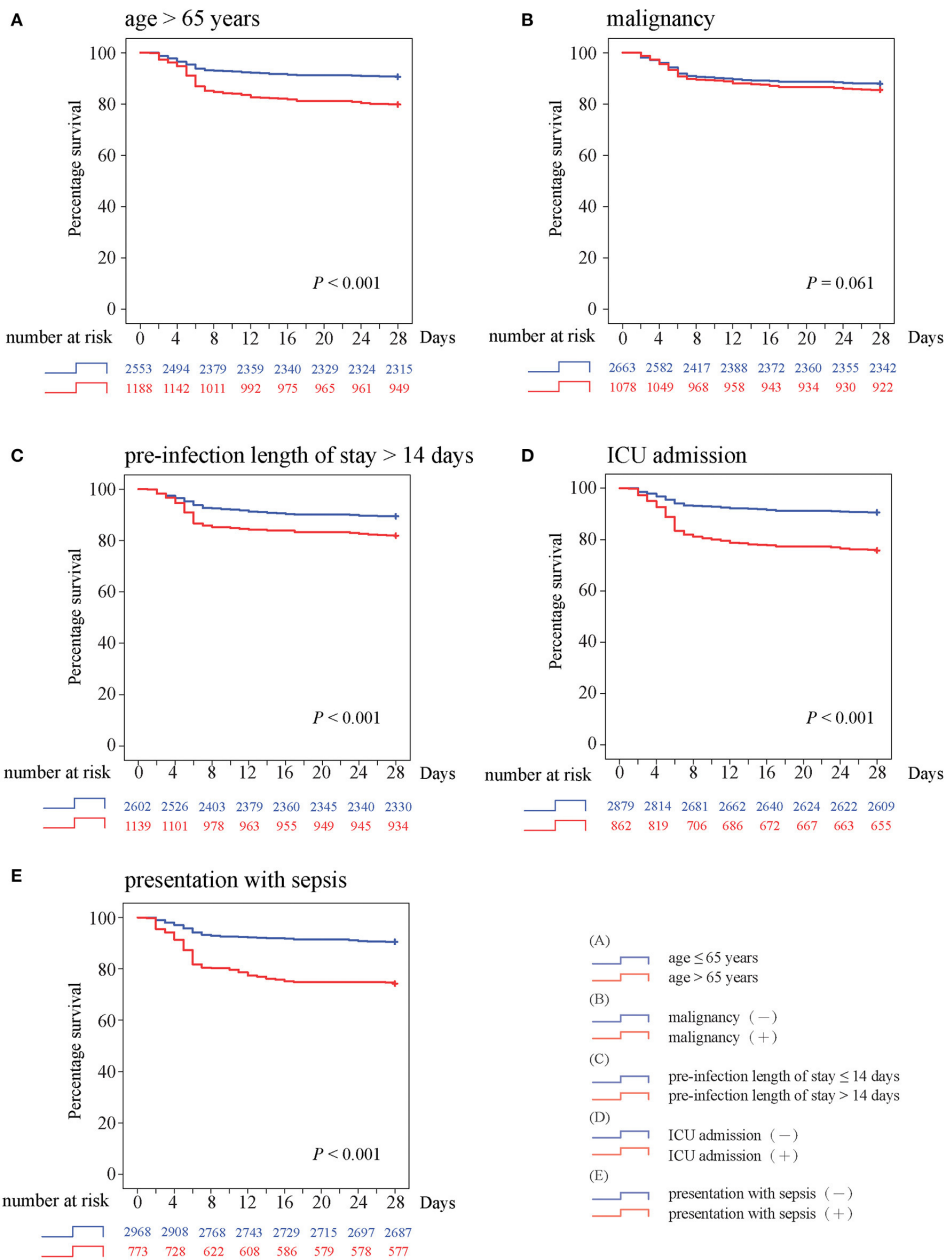
Kaplan–Meier survival curves with log-rank test for **(A)** age >65 years, **(B)** malignancy, **(C)** preinfection length of stay >14 days, **(D)** ICU admission, and **(E)** presentation with sepsis.

To evaluate the trends of in-hospital mortality, five predictors identified both in multivariate regression analysis and random forest predictive model were selected to construct survival curve analysis (Figure 4). Kaplan–Meier curves demonstrated that 28-day survival distributions were significantly different in patients with age >65 years (*P* < 0.001), pre-infection length of stay >14 days (*P* < 0.001), ICU admission (*P* < 0.001), and presentation with sepsis (*P* < 0.001). Although BSI patients with malignancy tended to have a worse outcome, the log-rank test was not significant (*P* = 0.061) and the two survival curves crossed early at around 4 days. All survival curves run parallel until the first week and start to diverge, with a continuously higher death rate among patients with corresponding prognostic factor.

## Discussion

Globally, the incidence of bacteremia remains high and continues to contribute to increased patient morbidity and mortality, as well as medical costs (1). In this study, a total of 4,708 BSI cases were obtained from the well-studied nationwide dataset over a 10-year period, and we reported that the 28-day all-cause mortality rate among hospitalized patients with BSI in China was 12.83%, which was slightly greater than that reported in a recent large multicenter study (12%) in the USA (18). Demographics, comorbidity, and clinical treatment information were investigated in our study to evaluate the predictors of mortality. The multivariate analysis showed that a total of five independent predictors for BSI mortality were identified in the dataset, which is associated with older age, malignancy, hospital length of stay, clinical symptom, and ICU admission. These predictive factors were also identified by a machine learning model and survival curve analysis.

Patients at increased risk of death after bacteremia could be identified in real time according to prognostic factors. Previous studies have reported that multiple clinical factors, including underlying medical conditions, previous antibiotics exposure, and severity of bacteremia, were independently associated with poor outcome in patients with BSI (19, 20). Patient-related factors, including older age, female sex, and recent hospitalization, were additional significant predictors of mortality (21). However, most of these studies focused on a specific subpopulation group suffering from BSI or those individuals infected with multidrug-resistant pathogen (21), while the current study extracted the predictive factors from a general patient population. The diverse population could increase the generalizability of the identified predictors. More complicated clinical manifestation could be available in the analysis, and these predictors might be more broadly applicable in clinical practice.

Previous studies proved that machine learning techniques are capable of harnessing a mass of clinical variables and the interaction between these factors and, ultimately, predicting clinical outcomes of interest with a satisfactory accuracy in real time (12). In the field of infectious disease prediction, the machine learning model has mostly been limited to the use of predicting infection with multidrug-resistant organism and sepsis in the ICU and emergency department (22–25). In this study, we made an attempt to utilize the machine learning prediction model to predict mortality among hospitalized patients with BSI, and it performed satisfactorily with an AUC value of 0.856. The prediction model also exported important predictors for BSI mortality, including ICU admission, presentation with sepsis, inappropriate empirical treatment, etc. These variables could aid in the physician's judgment and provide clinicians with real-time prognostic information to assist in decision-making and reduce preventable BSI-related adverse events. Moreover, no parameter optimization was performed for this model in order to simplify the application of machine learning approaches in the healthcare settings in our study. It suggests that parameter optimization could further improve predictive performance. In addition, other machine-learning-based models, such as the support vector machine, artificial neural networks, or deep learning, may also be constructed with this dataset and compared with the random forest model in this study.

Based on the nationwide culture-confirmed BSI cohort, we found that the overall mortality of BSI patients during the 10-year period was relatively stable, but the mortality of patients with BSI due to different causative pathogen presented different changing trends. *P. aeruginosa* and *A. baumannii*, two clinically important non-fermenters, were linked to increased mortality during the study period. Conversely, *S. aureus*-related mortality rate showed a gradually decreasing trend. A possible explanation for the observed phenomena was the extremely limited therapeutic options for bloodstream infections due to carbapenem-resistant *P. aeruginosa* and *A. baumannii* that have spread increasingly in recent years, while vancomycin- or daptomycin-resistant *S. aureus* were relatively rare in China.

In conclusion, our study determined the overall mortality rate of patients with bloodstream infection during a 10-year period and identified multiple predictors associated with poorer outcomes using multivariable-adjusted analysis and random forest predictive model. These clinically important predictive factors, including abnormal body temperature, longer hospital stay, and presentation with sepsis, could aid clinicians in identifying patients at high risk of death and lead to timely medical interventions to improve patient outcomes.

## Data Availability Statement

The original contributions presented in the study are included in the article/supplementary material, further inquiries can be directed to the corresponding author/s.

## Ethics Statement

The studies involving human participants were reviewed and approved by Research Ethics Board at Peking University People's Hospital. Written informed consent for participation was not required for this study in accordance with the national legislation and the institutional requirements.

## Author Contributions

LJ and HW conceived and designed the study. CZ, HL, RW, and QW led the data collection and analysis. LJ wrote the manuscript. HW critically reviewed and edited the manuscript. All authors have read, commented, and approved the final version of the article.

## Conflict of Interest

The authors declare that the research was conducted in the absence of any commercial or financial relationships that could be construed as a potential conflict of interest.
